# Emergence and Spreading Potential of Zika Virus

**DOI:** 10.3389/fmicb.2016.01667

**Published:** 2016-10-20

**Authors:** Álvaro Fajardo, Juan Cristina, Pilar Moreno

**Affiliations:** Laboratorio de Virología Molecular, Centro de Investigaciones Nucleares, Facultad de Ciencias, Universidad de la RepúblicaMontevideo, Uruguay

**Keywords:** ZIKV, emerging infectious diseases, genetic variability ZIKV, molecular evolution, spreading potential ZIKV

## Abstract

Zika virus (ZIKV) is an arthropod-borne *Flavivirus* (family *Flaviviridae*) closely related to dengue, yellow fever and West Nile viruses. ZIKV remained neglected, confined to enzootic transmission cycles in Africa and Asia, until the first significant outbreak was reported in Micronesia in 2007. Subsequent epidemics of growing incidence occurred in French Polynesia and other South Pacific Islands, and recently, in the Americas. The latter and currently ongoing outbreak of unprecedented incidence revealed the association of ZIKV infection with the occurrence of severe congenital malformations and neurological diseases, leading to a widespread concern about its potential to pose a global public health threat. Serological and molecular data suggest that the genetic and geographic diversification of ZIKV may be greatly underestimated. Here we discuss several ecological and epidemiological aspects, together with the evolutionary processes that may have driven the emergence and abrupt spread of ZIKV in the Americas.

## Introduction

Zika virus (ZIKV) is a mosquito-borne virus that belongs to the Spondweni serocomplex in the *Flavivirus* genus of the *Flaviviridae* family. ZIKV is closely related to dengue virus (DENV), yellow fever virus (YFV), and West Nile virus (WNV) (Kuno et al., 1998). Although ZIKV enzootic activity was repeatedly observed in different regions of Africa and Asia, significant human outbreaks have only recently occurred, in particular during current American epidemics (Haddow et al., 2013). Its high variability, great adaptability to vectors and hosts, as well as its association with neurological diseases and fetus malformations, has converted ZIKV in one of the biggest challenges for global health as regards to prevention, detection and prospect for control.

## Epidemiology

Zika virus was first detected in 1947 in rhesus monkeys, during a yellow fever routine surveillance in the Zika Forest in Uganda, where one year later was also isolated from a pool of *Aedes africanus* mosquitoes (Dick et al., 1952). The first human disease related with ZIKV was reported in 1954 in Nigeria (Macnamara, 1954). ZIKV enzootic activity was repeatedly observed in different regions of Africa and Asia, but only 14 human cases were reported until 2007 (Faye et al., 2014), when the first significant ZIKV outbreak occurred in Yap Island (Micronesia), leading to infection of 73% of the residents older than 3 years (Duffy et al., 2009). Later on, in 2013, a huge epidemic event occurred in French Polynesia with around 30.000 symptomatic cases that included low fever, maculopapular rash, arthralgia and conjunctivitis (Musso et al., 2014). ZIKV outbreaks were subsequently reported in different Pacific islands including New Caledonia, Solomon Islands, Cook Islands, Fiji, Samoa, Vanuatu, and Easter Island (Cao-Lormeau et al., 2014; Dupont-Rouzeyrol et al., 2015; Musso and Gubler, 2016). In March 2015, ZIKV was reported in Salvador, Brazil (Campos et al., 2015; Zanluca et al., 2015), and rapidly spread throughout the Americas, leading to autochthonous cases in 40 countries of this continent by July 7th, 2016 (PAHO, 2016).

Zika virus ZIKV infections in humans are mainly asymptomatic, causing in some patients a mild, self-limited febrile illness that can be accompanied by other clinical symptoms like rash, arthralgia or conjunctivitis (Simpson, 1964). This was the usual clinical picture until the French Polynesian outbreak in 2013, when severe neurological complications were observed. Epidemiological data from this outbreak documented a 20-fold increase from expected in the incidence of Guillain-Barré syndrome (GBS) (Oehler et al., 2014), as well as meningoencephalitis (Carteaux et al., 2016) and acute myelitis (Mécharles et al., 2016) cases. GBS was also associated with ZIKV infections during current epidemics in the Americas in 12 countries (Dirlikov et al., 2016; dos Santos et al., 2016; PAHO, 2016; Rozé et al., 2016). In addition, the latter outbreak coincided both in time and geographic location with an increase in the number of infants born with microcephaly and other central nervous system malformations (Schuler-Faccini et al., 2016; Ventura et al., 2016). By July 2016, 1.638 cases of congenital syndrome associated with ZIKV infection were confirmed in Brazil, with reported cases of this disorder in Colombia, El Salvador, French Guiana, Martinique, Panama, Puerto Rico, and United States (PAHO, 2016). Subsequent retrospective studies also revealed congenital cerebral malformations in newborns during French Polynesian outbreak of 2013 (Besnard et al., 2016; Cauchemez et al., 2016). Accumulating evidence supports the association between ZIKV infection and birth defects, including the detection of ZIKV RNA, viral particles, and/or viral antigens in placenta, amniotic fluid and fetal tissues, being the latter studies performed on microcephalic fetuses after miscarriage or neonatal death (Calvet et al., 2016; Ladhani et al., 2016; Martines et al., 2016; Meaney-Delman et al., 2016; Mlakar et al., 2016; Oliveira Melo et al., 2016; Rasmussen et al., 2016; Sarno et al., 2016). Moreover, a recent study reports the detection of anti-ZIKV IgM antibodies in cerebrospinal fluids of 30 out of 31 neonates with microcephaly, strongly suggesting a congenital infection with ZIKV (Cordeiro et al., 2016). In addition, experimental data indicated that ZIKV infects human neural progenitor cells attenuating their growth (Hughes et al., 2016; Li et al., 2016; Nguyen et al., 2016; Tang et al., 2016).

As previously discussed, until 2007 our knowledge of this ZIKV was restricted to limited confirmed cases in Africa and Asia. However, on the basis of entomological, epidemiological and seroprevalence studies, it can be deduced that the incidence, prevalence and dispersion of ZIKV have been significantly underestimated (Musso and Gubler, 2016). Human serosurveys suggest that ZIKV might be endemic in most part of Africa and South–East Asia (Dick et al., 1952; Smithburn, 1952, 1954; Hammon et al., 1958; Pond, 1963; Fagbami, 1979; Petersen et al., 2016), although it is important to note that the specificity of the serological studies used is uncertain due to the significant cross-reaction between different flaviviruses (Lazear and Diamond, 2016). This silent circulation may be explained by the fact that most ZIKV infections are asymptomatic, and clinical manifestations are generally mild and can be mistaken with other arboviral infections, leading to significant misdiagnosis and underreporting (Haddow et al., 2012). For instance, patients of Micronesian outbreak of 2007 were initially diagnosed with dengue fever (Lanciotti et al., 2008; Duffy et al., 2009). The same happens in most tropical and subtropical regions where other non-specific diseases like dengue and/or chikungunya are endemic (Nhan and Musso, 2015). Moreover, as ZIKV is not commonly tested in routine diagnostic assays, a considerable number of cases are expected to remain undetected. For example, recently retrospective analyses revealed a widespread distribution of ZIKV in Thailand (Buathong et al., 2015), as was previously suggested by the confirmation of imported cases from travelers returning from that country (Fonseca et al., 2014; Tappe et al., 2014). Several other reports of imported cases from tourists that have visited different Asian and South Pacific Islands reveal that circulation of ZIKV remain silent in different countries (Deng et al., 2016; Korhonen et al., 2016; Zhang et al., 2016).

## Transmission

Zika virus circulation has been mainly reported in sylvatic enzootic transmission cycles, involving arboreal mosquitos and non-human primates (Darwish et al., 1983; Hayes, 2009; Faye et al., 2014). Several mosquitoes species have been related with African and Asian jungle cycles, especially *A. africanus* (Dick et al., 1952; Haddow et al., 1964; Berthet et al., 2014; Diallo et al., 2014), as well as other *Aedes spp.* mosquitoes (Marchette et al., 1969; Cornet et al., 1979b; Fagbami, 1979; McCrae and Kirya, 1982; Akoua-Koffi et al., 2001; Berthet et al., 2014; Diallo et al., 2014). In enzootic transmission cycles, humans and other mammals may act as occasional dead-end hosts (Kenney and Brault, 2014). ZIKV antibodies have been detected in several vertebrates (Darwish et al., 1983; Haddow et al., 2012), suggesting that other animals may be involved in natural transmission cycles.

Humans accidentally infected may potentially act as hosts leading to urban cycles, if they exhibit high and sustainable viremia (Kuno and Chang, 2005; Duffy et al., 2009). The role of a bridge vector between both ecologically distinct transmission cycles is essential for these events to occur. Several mosquito species have been studied in terms of their host choice to evaluate their potential as bridge vectors of different arboviruses (Kaddumukasa et al., 2015). *A. vittatus* have been suggested to link both ZIKV cycles in Africa (Diallo et al., 2014). *A. albopictus* was found to be implicated in DENV cycle switching in Asia (Vasilakis et al., 2011; Hanley et al., 2013), and therefore, it is likely to play the same role with ZIKV. Moreover, experimental data have shown *A. albopictus* adaptability to transmit ZIKV (Wong et al., 2013; Chouin-Carneiro et al., 2016; Di Luca et al., 2016), as has been observed at least in Gabon in 2007 (Grard et al., 2014). However, *A. aegypti* seems to have the highest vectorial capacity in urban cycles (Lord et al., 2015), and has been related with most human outbreaks, including current American epidemics (Boorman and Porterfield, 1956; Marchette et al., 1969; Cornet et al., 1979a; Olson et al., 1981; Li et al., 2012; Weaver et al., 2016). Both *A. aegypti* and *A. albopictus* are anthropophilic mosquitoes that are widely distributed throughout tropical and subtropical regions and are also competent vectors for other arboviruses like DENV, YFV and chikungunya virus (CHIKV) (Musso and Gubler, 2016). Other mosquito species, like *A. hensilli* and *A. polynesiensis*, have been, respectively, responsible for ZIKV outbreaks in Yap Island in 2007 (Ledermann et al., 2014) and French Polynesia in 2013–2014 (Musso et al., 2014), suggesting that the potential role of other *Aedes spp.* as additional vectors should not be ruled out.

Although mosquito-borne transmission is the most common route for ZIKV infection, current South Pacific/American outbreaks revealed other modes of biological transmission. In particular, the elevated number of newborns with microcephaly raised concern about maternal-fetal transmission during pregnancy. As previously discussed, growing evidence support trans-placental transmission, and perinatal transmission of ZIKV has also been reported in French Polynesia (Besnard et al., 2014). Furthermore, recent studies have demonstrated that ZIKV RNA can be found in semen 62 and 93 days after onset of symptoms, although virus infectivity was not tested as cell culture assays were not performed (Atkinson et al., 2016; Mansuy et al., 2016). This finding is in line with the amount of sexually transmitted cases that have been lately reported (Musso et al., 2015; Deckard et al., 2016; D’Ortenzio et al., 2016; Frank et al., 2016; Hills et al., 2016; Turmel et al., 2016; Venturi et al., 2016). Moreover, recently published data suggest the first case of female to male sexual transmission (Davidson et al., 2016). Despite the potential of sexual transmission to promote an epidemic has been predicted to be unlikely (Yakob et al., 2016), these findings indicate that this route of transmission may contribute to dispersal of ZIKV greater than initially thought.

## Genetic Variability of ZIKV

**FIGURE 1:**
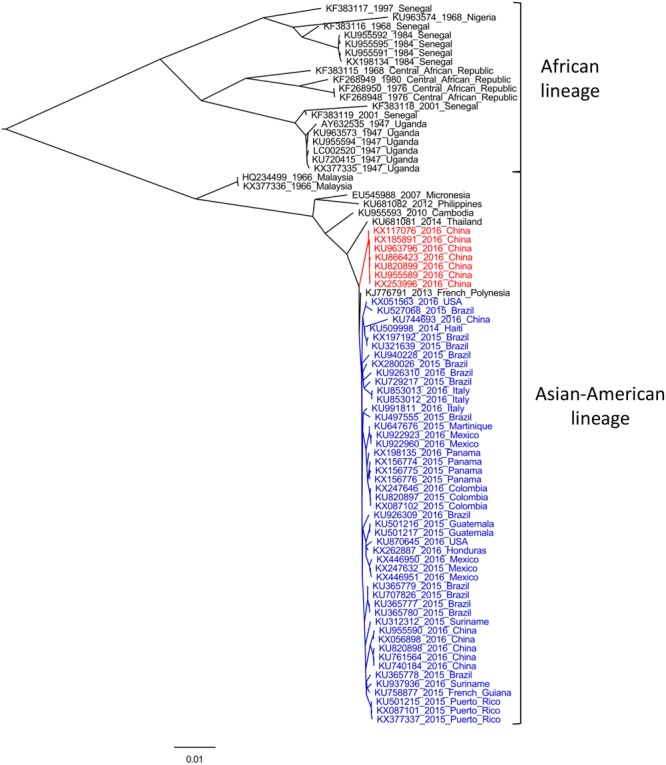
**Maximum-likelihood analysis of all complete ORF sequences of Zika virus (ZIKV) available.** The phylogenetic tree was performed using PhyML software (Guindon et al., 2005) under a GTR+G+I substitution model, and edited with FigTree program v1.4.2 (available at: http://tree.bio.ed.ac.uk). Strains are indicated by their accession number, year and country of isolation. Sequences derived from variants isolated during current American outbreak and from imported strains from Fiji and Samoa (Deng et al., 2016; Zhang et al., 2016), are indicated in blue and red, respectively. Bootstrap values >90% support major nodes but are not shown for visualization purpose. The bar at the bottom of the tree denotes nucleotide distance.

Zika virus is a single-stranded, positive sense, RNA virus with a genome of approximately 10,794 nt in length. Its genome carries a single open reading frame (ORF) that encodes three structural (capsid (C), precursor of membrane (prM) and envelope (E)) and seven non-structural (NS) proteins, with 2 flanking non-coding regions (5′ and 3′ NCR) (Kuno and Chang, 2007). Several phylogenetic analyses have been performed in order to investigate the genetic diversity of ZIKV, using both partial and complete genome regions (Lanciotti et al., 2008; Haddow et al., 2012; Faye et al., 2014; Enfissi et al., 2016; Fajardo et al., 2016; Faria et al., 2016; Gong et al., 2016; Shen et al., 2016; Wang et al., 2016). Full-length ORF sequences analyses reveal that the high error rate of RNA-dependent RNA polymerase has driven ZIKV to evolve into two different genetic groups, denominated African and Asian-American lineages (see **Figure 1**) (Haddow et al., 2012; Enfissi et al., 2016; Faria et al., 2016).

African lineage comprises strains isolated in Burkina Faso, Central African Republic, Cote d’Ivoire, Gabon, Nigeria, Senegal, and Uganda, and can be further divided in two sub-lineages, as was indicated by phylogenetic approaches restricted to RNA-polymerase (NS5) (Lanciotti et al., 2008) or E (Faye et al., 2014) coding regions. On the basis of phylodynamic studies, Faye et al. (2014) suggested that the most common recent ancestor of all reported ZIKV strains circulated around 1920 in Uganda, from where it spread to West Africa and afterward to Malaysia, giving rise to Asian (currently Asian–American) lineage (Faye et al., 2014). This genetic group has been better characterized, especially after current epidemics when several full-length sequences were obtained. This lineage clusters together ancestral Malaysian strains (1966) with variants from Micronesia (2007), Cambodia (2010), Philippines (2012), Thailand (2014), and all ZIKV strains reported in the ongoing American outbreak (**Figure 1**, in blue), which are closely related to French Polynesian variants of ZIKV epidemics of 2013. Recent studies indicated that currently ZIKV strains circulating in the Americas emerged from a single introduction of an ancestor that existed in French Polynesia between August 2013 and June 2014 (Fajardo et al., 2016; Faria et al., 2016). This hypothesis is in line with observed phylogenetic patterns, as French Polynesian variant roots the American cluster (**Figure 1**). However, seven variants recently obtained from Chinese travelers returning from Fiji and Samoa (**Figure 1**, in red), share a different evolutionary history, revealing that two different sub-lineages are responsible of current ZIKV epidemics in the Americas and in South Pacific Islands (Deng et al., 2016; Zhang et al., 2016). Moreover, a recent study indicated that these imported Chinese strains share an ancestor with American variants that circulated around May 2013 (Fajardo et al., 2016), coinciding with the time when the ancestor of all American isolates was circulating (Faria et al., 2016). This finding indicates that two different evolutionary routes were followed by ancestral strains that emerged in French Polynesia, giving rise to different contemporaneous sub-lineages circulating in the Americas and in South Pacific Islands, revealing that ZIKV diversification may be greatly underestimated (Fajardo et al., 2016).

Unfortunately, there are no other full-length sequences available from the Pacific Islands outbreak, which limits our interpretation of the evolutionary patterns, diversity and pathogenicity of currently circulating ZIKV strains. In fact, the lack of complete genome sequences distributed over time and space has been one of the major historical limitations to explore in detail the phylogenetic relationships of ZIKV variants and their spatio-temporal distribution. For instance, recent reports have suggested that another genetic group, African lineage II, is revealed when phylogenetic relationships are deduced through the analysis of E and NS5 coding regions (Gong et al., 2016; Shen et al., 2016). This lineage group together one variant of Cote d’Ivoire of 1980 and six strains isolated in Senegal between 1998 and 2001 (Faye et al., 2014). Furthermore, both African and Asian-American clusters seem to be rooted by this lineage, which also suggests a greater ZIKV genetic and geographic diversification than expected (Shen et al., 2016). This hypothesis was also supported by Gong et al., who calculated a genetic distance of 0.212 substitutions/site between the E gene of two contemporary isolates of Senegal, belonging to African lineages I and II, respectively. Considering evolutionary rates of the order of 10^-3^ substitutions/site/year, as recently estimated for American ZIKV strains (Fajardo et al., 2016; Faria et al., 2016), ZIKV may have been circulating in Africa for more than 100 years (Gong et al., 2016). Therefore, the genetic characterization of variants of this lineage would provide essential information to investigate the origin, geographic dispersion and genetic diversity of ZIKV.

## ZIKV Spreading Potential

The emergence and dispersal of ZIKV in the Americas reminds us of the route that was previously followed by other arboviruses, like DENV and CHIKV, which were first detected in Africa, and spread subsequently to Asia and the Americas. This migration pattern is associated with ZIKV capacity to adapt to urban vectors, like *A. aegypti*, which allows its expansion into human environments. A general characteristic that is shared between different arboviruses is their high mutation rates, which provide them with the possibility to explore different phenotypical changes in their continually evolving process to adapt to different vectors and hosts (Kuno and Chang, 2005). For instance, a single amino acid change in its surface glycoprotein allows CHIKV to switch its competent vector to *A. albopictus* (Tsetsarkin et al., 2007). Therefore, genetic changes play a major role in adaptation of arboviruses to different hosts and vectors, as has been also reported in DENV (Messer et al., 2003; Bennett et al., 2010) and WNV (Malkinson et al., 2002; Brault et al., 2007; Moudy et al., 2007).

The recent epidemics in South Pacific and the Americas with its unprecedented association with microcephaly and GBS cases, led to the proposal of different hypothesis to explain this emergence. It is likely that as a consequence of different genetic changes, ancestral Asian lineage gave rise to epidemic strains that become better adapted to humans, leading to infections with higher viremia levels, enhancing trans-placental transmission and modulating changes in cell tropism (Musso and Gubler, 2016; Weaver et al., 2016). Moreover, recent analyses have suggested a bias in the codon usage of ZIKV to increase its fitness in humans (Freire et al., 2015; Cristina et al., 2016; Russell, 2016). This hypothesis is supported by the short-term diversification observed among recent isolates (**Figure 1**), revealing the expansion of ZIKV into a large naïve population (Weaver et al., 2016). It has also been hypothesized that the rare congenital malformations and immunological disorders may be in fact low frequency events in ZIKV infections, that are now exposed due to the extent of recent outbreaks (Musso and Gubler, 2016). Furthermore, recent studies have suggested that pre-existing DENV antibodies may enhance ZIKV infection through an antibody-dependent enhancement mechanism, similar to what happens in secondary infections of DENV (Dejnirattisai et al., 2016; Paul et al., 2016). If this is confirmed it would have huge implications for disease pathogenesis, considering that current epidemics are occurring in regions where most of the population has already been exposed to DENV. Additionally, this mechanism of infection enhancement, together with ZIKV adaptability to vectors/hosts, the widely distribution of competent mosquitoes and the unusual non-vectorial human-to-human transmission routes observed, can help to interpret the reasons for the suddenly explosive emergence of ZIKV in the Americas and its potential to spread into other geographic regions.

## Conclusions

Current ZIKV epidemics occurring throughout the American continent represent the most recent example of a mosquito-borne virus introduction into previously unaffected areas with immunologically naïve population. This unexpected emergence follows recent arrival and spreading of DENV, WNV, and CHIKV, which clearly responds to human activities that promotes optimal ecological environments for vectorial activity. Although ZIKV has remained almost ignored for half a century, entomological, epidemiological and molecular studies have strongly indicated that its incidence, geographic dispersion and genetic diversity have been significantly underestimated. Its adaptability to different mosquito species allowed ZIKV to spread into efficient urban transmission cycles helped by the widespread distribution of competent vectors. Furthermore, unprecedented routes of transmission for flaviviruses, including maternal-fetal and sexual intercourse, may contribute to increase its spreading potential.

## Author Contributions

AF, JC, and PM contributed to the elaboration or this minireview. All authors read and approved the final manuscript.
